# Transcriptional Regulatory Networks in Hepatitis C Virus-induced Hepatocellular Carcinoma

**DOI:** 10.1038/s41598-018-32464-5

**Published:** 2018-09-24

**Authors:** Marwa Zahra, Hassan Azzazy, Ahmed Moustafa

**Affiliations:** 1 Biotechnology Graduate Program, American University, New Cairo, 11835 Egypt; 2 Department of Chemistry, The American University in Cairo, School of Sciences & Engineering, New Cairo, 11835 Egypt; 3 Department of Biology, The American University in Cairo, New Cairo, 11835 Egypt

## Abstract

Understanding the transcriptional regulatory elements that influence the progression of liver disease in the presence of hepatitis C virus (HCV) infection is critical for the development of diagnostic and therapeutic approaches. Systems biology provides a roadmap by which these elements may be integrated. In this study, a previously published dataset of 124 microarray samples was analyzed in order to determine differentially expressed genes across four tissue types/conditions (normal, cirrhosis, cirrhosis HCC, and HCC). Differentially expressed genes were assessed for their functional clustering and those genes were annotated with their potential transcription factors and miRNAs. Transcriptional regulatory networks were constructed for each pairwise comparison between the 4 tissue types/conditions. Based on our analysis, it is predicted that the disruption in the regulation of transcription factors such as *AP-1*, *PPARγ*, and *NF-κB* could contribute to the liver progression from cirrhosis to steatosis and eventually to HCC. Whereas the condition of the liver digresses, the downregulation of miRNAs’ (such as miR-27, Let-7, and miR-106a) expression makes the transition of the liver through each pathological stage more apparent. This preliminary data can be used to guide future experimental work. An understanding of the transcriptional regulatory attributes acts as a road map to help design interference strategies in order to target the key regulators of progression of HCV induced HCC.

## Introduction

HCV is an epidemic affecting an estimated 160 million individuals worldwide^1^. At the onset of HCV infection, the liver begins to react to the virus in many ways. As the virus begins to replicate, the liver sends signals to genes whose expression is stimulated by interferon, interferon-stimulated genes (ISGs) to react to these foreign particles in the body. For the majority of cases, HCV digresses the condition of the liver from cirrhosis, to steatosis, and eventually carcinoma.

Chronic HCV can lead to cirrhosis which may develop rapidly; within 1–2 years of exposure, or slowly within 2–3 decades. In studies with 10–20 years of follow-up, cirrhosis develops in 20–30 percent of patients^2^. Once cirrhosis develops, symptoms of end-stage liver disease can appear. Another condition that can hasten the development of cirrhosis in the liver and lead to the progression of end-stage liver disease in the presence of HCV infection is hepatic steatosis. Hepatic steatosis, excessive lipid accumulation within the hepatocyte cytoplasm, has been recognized as the sole route for a direct cytopathic effect by the HCV^3^. Several mechanisms have been proposed as to the process of lipid accumulation in the liver such as; HCV core protein may be interacting with apolipoprotein^4^. Also, it has been suggested that the HCV core protein induces oxidative stress within the mitochondria which leads to or contributes to lipid accumulation^5^. Hepatic steatosis leads to the progression of end-stage liver disease, whether steatosis achieves this by aggravating fibrosis itself or factors causing steatosis are also aggravating fibrosis is still unknown.

While IFN treatment may reduce the incidence of HCC, it is an inevitable event as the liver digresses by steatosis and cirrhosis. The mechanism by which this eventual digression leads to carcinoma is still unknown. However, through analysis of the key components that lead to the progression of liver disease to carcinoma, it is apparent that the condition of the liver is at large the reason behind the inability of the liver to maintain cellular homeostasis and then eventually digresses to carcinoma^6^.

The development of HCV-induced HCC is progressive and may occur over a period of 20–40 years (ref.). This multi-step progression involves establishment of chronic HCV infection, chronic hepatic inflammation, progressive liver fibrosis, initiation of neoplastic clones accompanied by irreversible somatic genetic/epigenetic alterations, and progression of the malignant clones in a carcinogenic tissue microenvironment^7^. Yet, each step of HCV-induced hepato-carcinogenesis is a potential target for therapeutic intervention or chemoprevention. Because, HCV is a RNA virus with limited integration of its genetic material with the host’s genome, the carcinogenic potential of HCV is generally assumed to be linked to indirect mechanisms. A major obstacle for the understanding of the mechanisms linking HCV infection, inflammation and carcinogenesis is the lack of an *in vitro* and *in vivo* model systems^8^.

Systems biology approach studies biological systems by systematically breaking them down and looking at the gene, protein, and informational pathway responses. The integration of this data by means of mathematical models ultimately describes the structure of the system, and in turn answers questions that may arise to any disruptions that may happen to that system^9^. New technologies for systematically characterizing cellular responses have brought about this new approach to understanding biological systems. Tools such as DNA sequencers, microarrays, and high-throughput proteomics have become the methods of choice for rapid and comprehensive assessment of biological system properties and dynamics. Microarrays are a powerful tool that allows interrogation of known human transcriptomes. This increases the capacity for a suitable diagnostic approach to many diseases and a more refined method for approaching suitable treatment.

Using a system approach to study HCV-induced HCC, allows assessment of the individual genes involved as well as the regulatory aspects of those genes. This can be used for the construction of transcription regulatory networks which takes into account all the regulatory elements which focus on a particular gene of interest. In these networks, transcription factors’ (TFs) databases such as TRANSFAC, JASPAR, and SWISSREGULON are utilized to obtain information about the binding motif sites related to these TFs, as well as the potential target genes. Also, understanding how HCV affects biological systems would not be complete without considering miRNAs. miRNAs are known to regulate gene transcription via the silencing target mRNA transcripts. Over 2500 mature human miRNA have been isolated and described in a public database known as miRBase^10^. Therefore, understanding the changes in gene transcription regulated by miRNAs and provoked by HCV may explain causes for fibrosis and carcinoma in liver tissues.

Since, HCV attacks the liver in a manner that disrupts its normal functioning, it would be important to know where, in the regulation of normal functioning, this disruption takes place. Accordingly, this study places an emphasis on the study of the interactions that take place in the transcription regulation that alters gene expression in HCV induced HCC.

## Results

### Determining Differentially Expressed Genes

#### Transcription Regulatory Networks in HCV-induced HCC

The 124 samples used in this study were obtained from the Gene Expression Omnibus (GEO) with the identification number GSE14323. This dataset was used in particular because it provided a sample pool that would allow for a distinct look at the transcriptional control of genes^11^. It was also the best publicly available dataset that provided a clear stage by stage tissue sample for analysis and comparison^11^.

These samples were grouped into four sample clusters based on the tissue type from which they originated. The source of the microarray data had five types of samples in which the fifth was a group of 3 patients that provided cirrhotic or tumor tissue. This fifth sample type was incorporated into one of the four tissue types because it was not a clear stage but rather one of the other four stages already present. Sample clusters were then analyzed using R studio in order to determine differential expression of genes in each of the 4 distinct samples’ clusters.

There was an obvious high correlation between the means of expression values for genes in the cases of Cirrhosis and Cirrhosis with HCC. Yet, the HCC samples showed to be more closely correlated to Cirrhosis Samples but not Cirrhosis HCC. Normal tissue samples showed the least correlation between Cirrhosis samples and Cirrhosis HCC samples, and showed more of a correlation with HCC samples in the mean expression values of genes.

In order to further analyze differential expression in each of the four clusters, they were compared against one another in the aforementioned six pairwise comparisons. For each pairwise comparison, a scatter plot and a volcano plot were generated using R studio in order to filter the differentially expressed genes with biological and statistical significance. These filtered genes that were found to have both biological and statistical significance were determined and further analyzed for their corresponding regulating transcription factors as well as miRNAs.

### Transcription Factors

Transcription factors for differentially expressed genes were determined using a series of databases and online resources. These transcription factors were further assessed for their frequency in regulating all of the differentially expressed genes between two conditions. In the Normal_Cirrhosis comparison, 12 transcription factors were found to regulate differentially expressed genes most frequently. These transcription factors include *AML1a*, *AP-1*, *ATF-2*, *c-Jun*, *CREB*, *C/ebpalpha*, *HNF-1*, *HNF-4alpha*, *PPAR-gamma*, *STAT3*, *STAT5*, *and NF-Kβ*.

In Fig. 2, a network was constructed showing those transcription factors and the differentially expressed genes that they regulate. In this comparison, it was also observed that the differentially expressed genes that had the greatest fold change were *TACSTD2*, *TRIM22*, *MGP*, and *CTGF*. However, in the pairwise comparison between Normal and HCC, the network had fewer number of edges showing a decrease in interaction between differentially expressed genes and transcription factors. In Fig. 3 from Normal to HCC, although the same 12 aforementioned transcription factors showed high expression there was a significant decrease in the number of differentially expressed genes regulated by these transcription factors.

**Figure 1:**
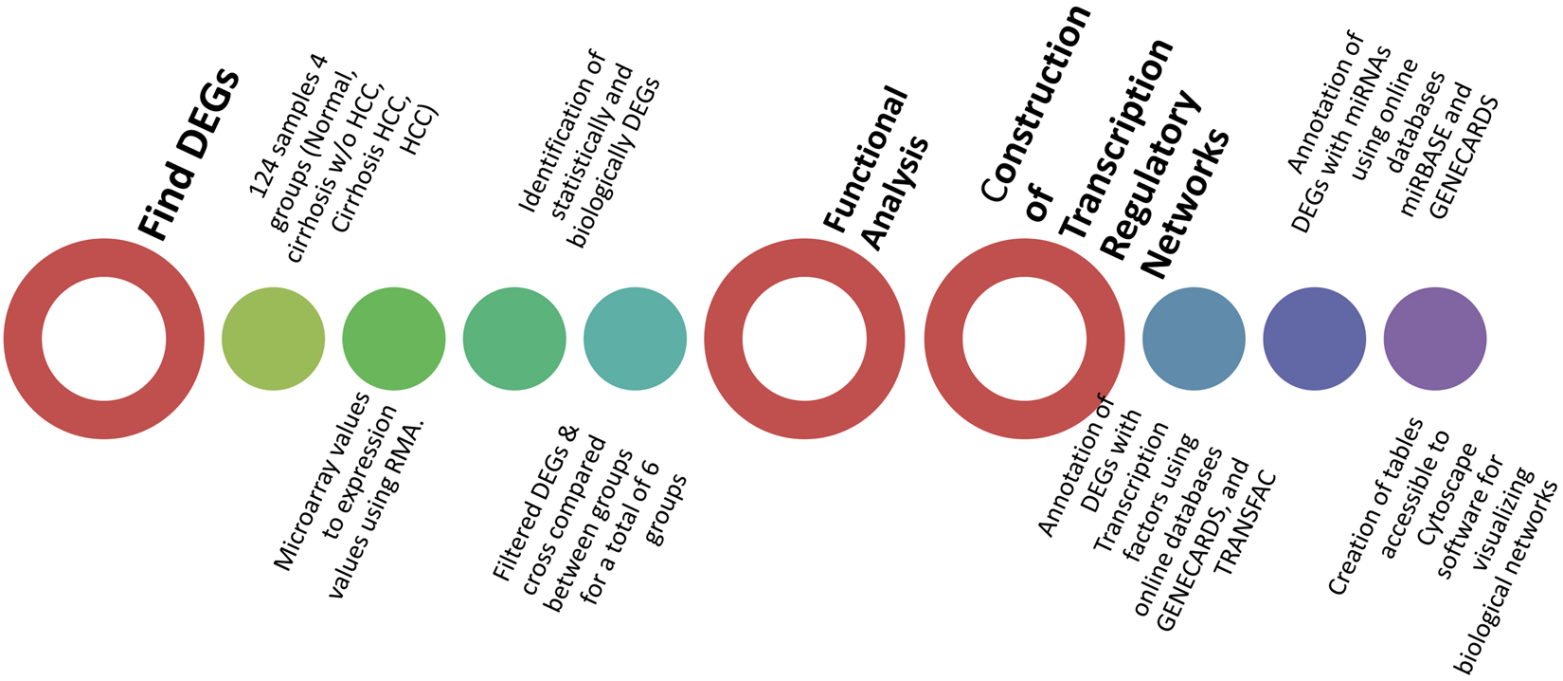
Schematic display of study design. As an overview of how this study was performed this diagram shows the process that the study proceeded in to create transcriptional regulatory networks for HCV-induced HCC.

**Figure 2:**
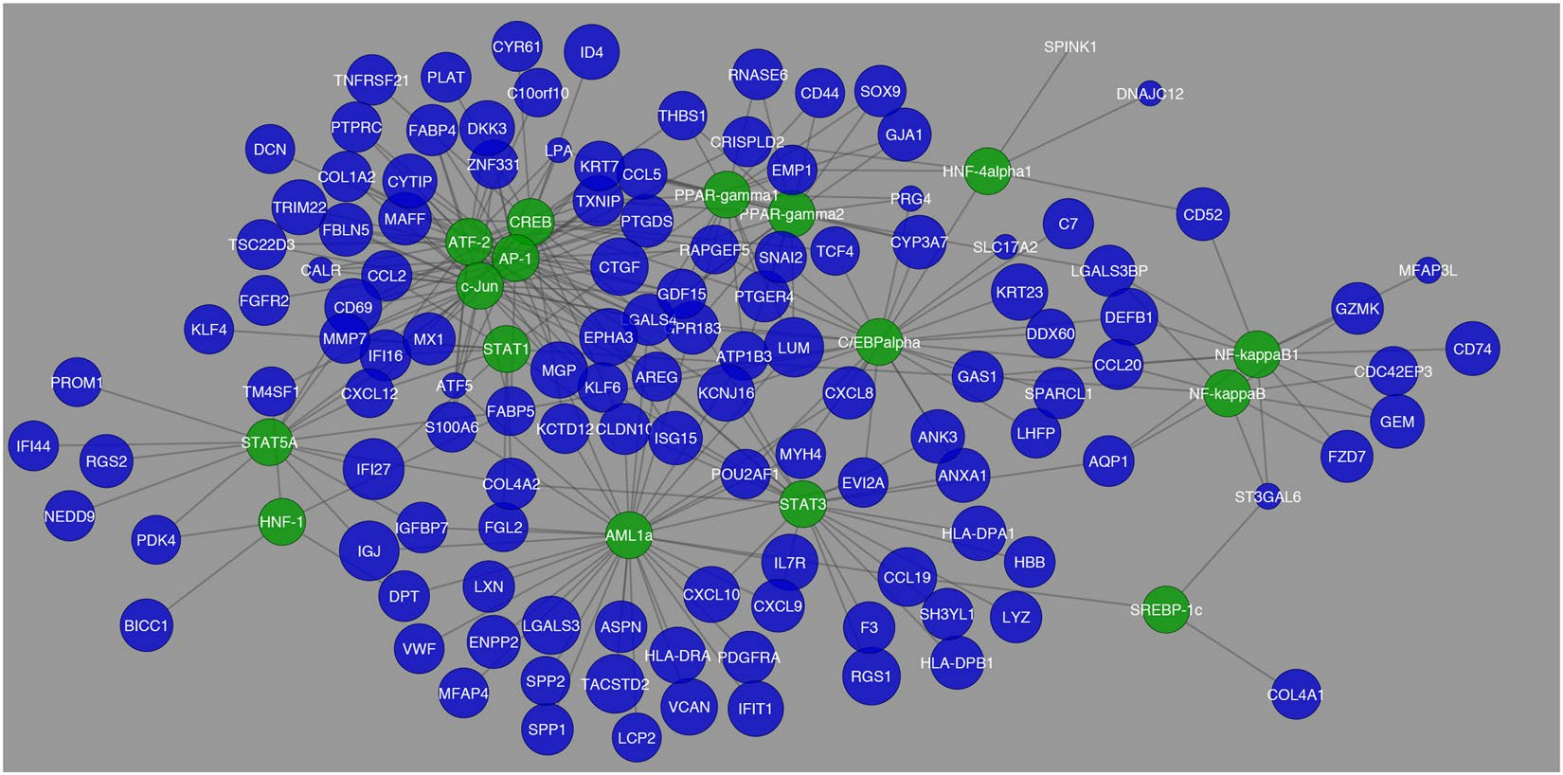
Network visualization of transcription factors that were found to be the most common in regulation of differentially expressed genes in the comparison between Normal vs. Cirrhosis. The blue circles represent differentially expressed genes and their respective size is representative of their fold change value. The green circles represent the transcription factors that are regulating their respective differentially expressed genes.

**Figure 3:**
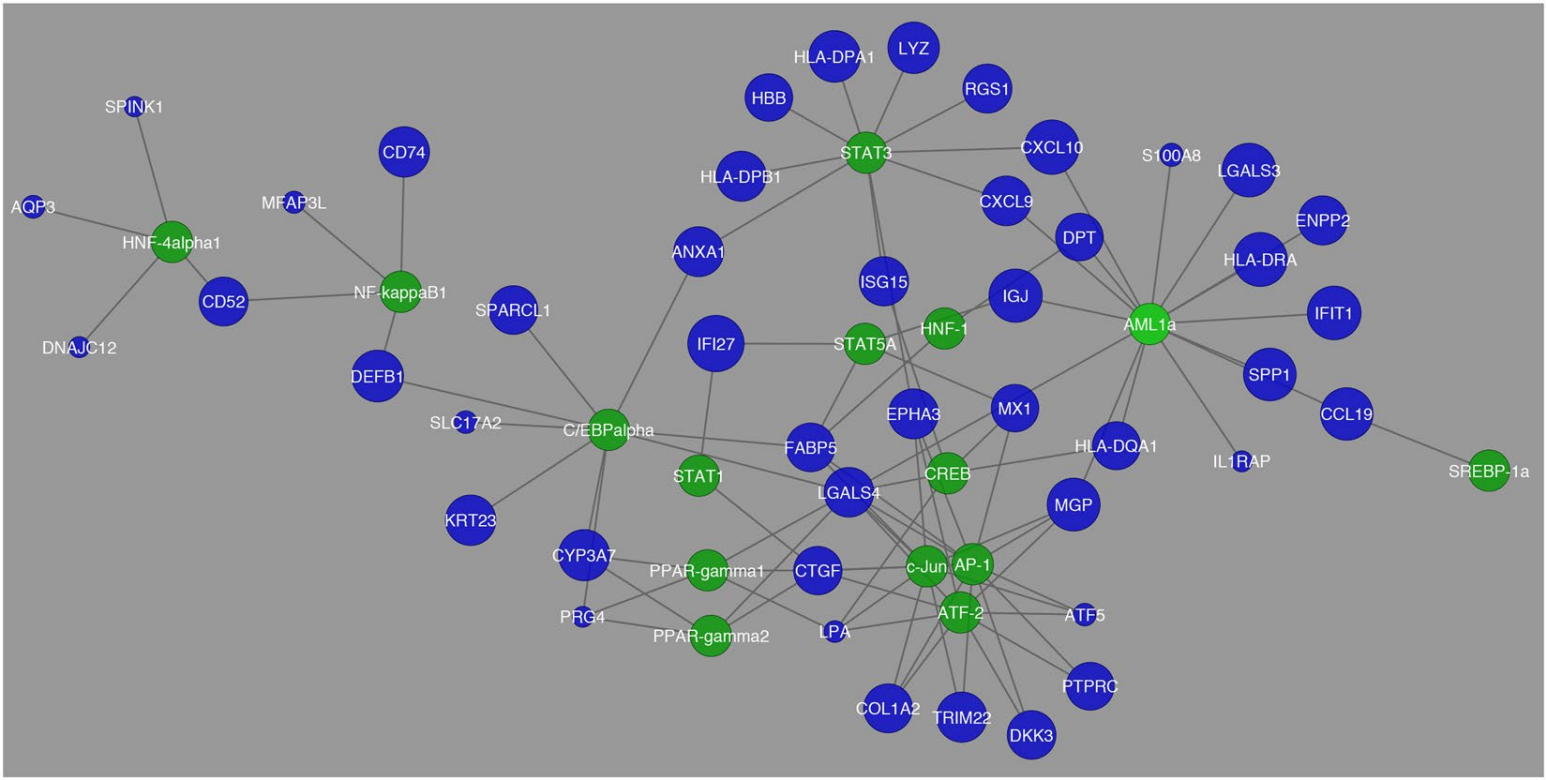
Network visualization of transcription factors that were found to be the most common in regulation of differentially expressed genes between Normal and HCC. The blue circles represent differentially expressed genes and their respective size is representative of their fold change value. The green circles represent the transcription factors that are regulating their respective differentially expressed genes.

**Figure 4:**
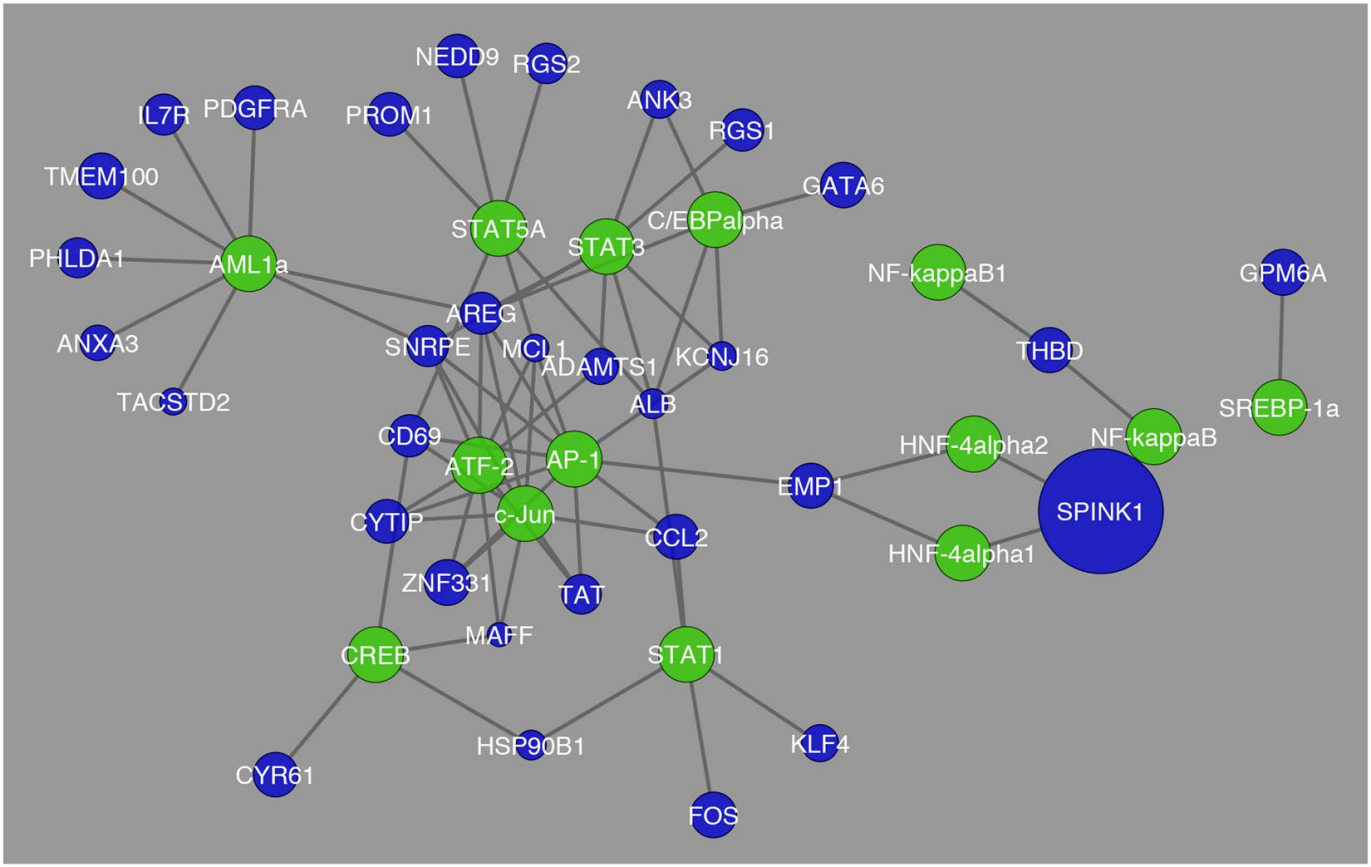
Network visualization of transcription factors that were found to be the most common in regulation of differentially expressed genes between cirrhosis and HCC. The blue circles represent differentially expressed genes and their respective size is representative of their fold change value. The green circles represent the transcription factors that are regulating their respective differentially expressed genes.

In this pairwise comparison, the differentially expressed genes that showed the greatest fold change were *IFI27*, *CXCL10*, *LGALS3*, *HLA-DRA*, and *IFIT1*. This was also different from what was observed before. The network analysis for the Cirrhosis vs. HCC displays more distinctly the interaction between the 12 transcription factors with highest expression and their regulated differentially expressed genes. This marks a designation between the previous comparisons in that it compares the two stages of a diseased liver and its transcriptional interaction. In Fig. 4 the interactions between transcription factors and differentially expressed genes becomes more distinct as well.

**Figure 5:**
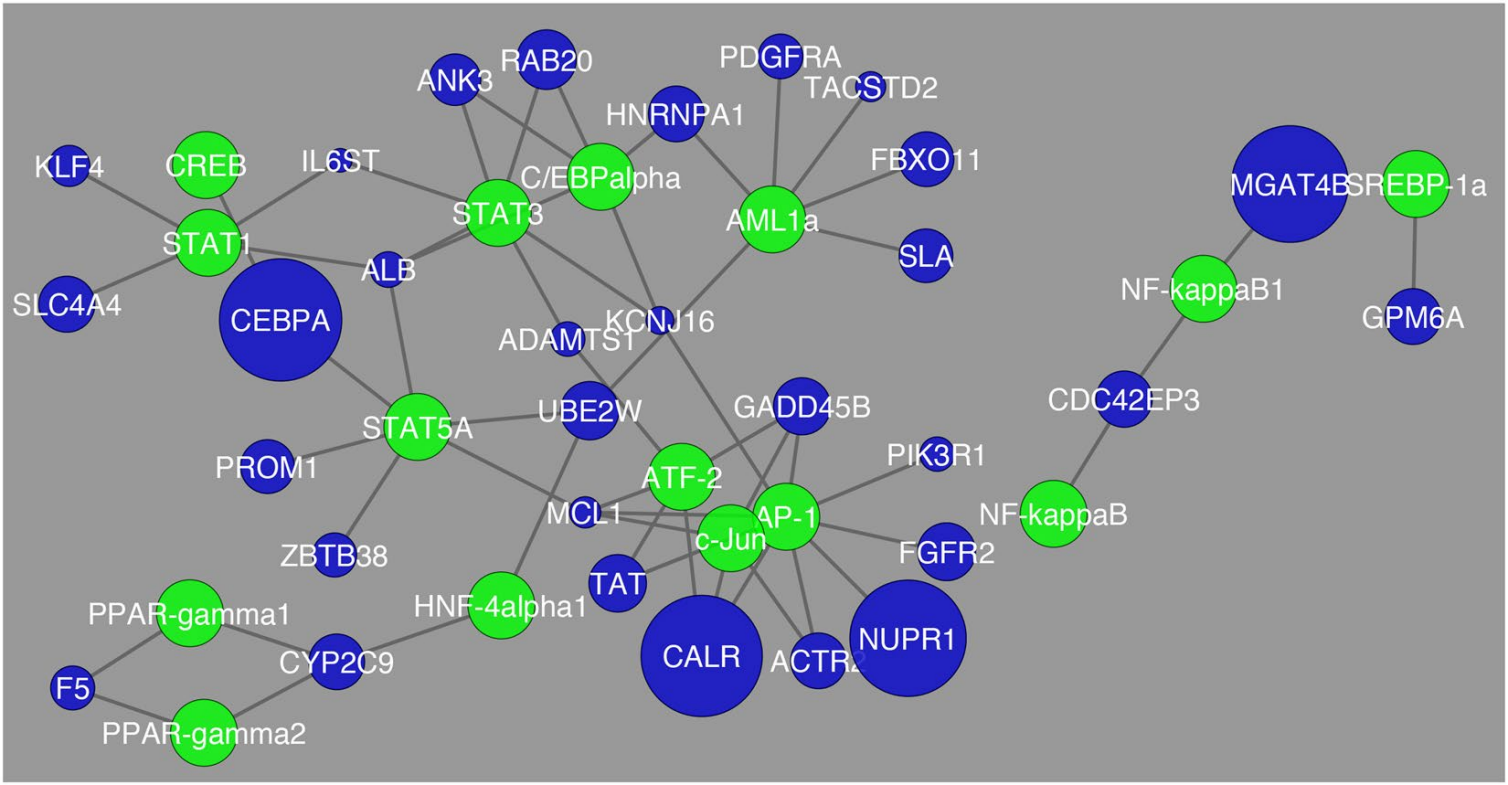
Network visualization of transcription factors that were found to be the most common in regulation of differentially expressed genes between cirrhosis HCC and HCC. The blue circles represent differentially expressed genes and their respective size is representative of their fold change value. The green circles represent the transcription factors that are regulating their respective differentially expressed genes.

In this pairwise comparison there was a single gene that showed a significant fold change as seen in Fig. 4 marked by its size and that is *SPINK1*. These findings are quite distinct from what was observed for the comparison between Cirrhosis HCC and HCC. In Fig. 5, the interactions between transcription factors in this comparison is different from what was observed before and there are more genes with greater fold change than in the previous comparison (Cirrhosis vs HCC). The genes that were determined to have the greatest fold change were *CEBPA*, *CALR*, *NUPR1*, and *MGAT4B*.

### MiRNAs

Another database (miRbase) was used in order to determine miRNAs that regulate differentially expressed genes^10^. This allowed for an annotation of differentially expressed genes with the miRNAs that regulate them. A plethora of potential miRNAs were found to regulate filtered differentially expressed genes for each pairwise comparison and due to the plethora of miRNA that are naturally found in the liver, miRNAs that were chosen for further analysis were those that showed significant differential expression pattern of genes that they regulated in the group. Also, a review of the literature made it possible to determine the type of regulation of these miRNAs observed in the presence of HCC. In Table 1, it becomes apparent that the majority of the miRNAs that are involved in regulation of differentially expressed genes are downregulated in the presence of HCC. The miRNAs in Table 1 were constructed in a network as a visualization of the genes that are involved with these miRNAs.

**Table 1:** Differentially expressed genes annotated with their potential miRNAs were analyzed for expression patterns of miRNAs.

miRNAs with highest Expression Patterns		
miRNA	Regulation in HCV-HCC	Reference
miRNA-335-5p	Up	^10^
miRNA-128	UP	^11^
miRNA-27 a&b	Down	^12^
miRNA-106a	Down	^12^
miRNA-15a	Down	^12^
miRNA-181 a&c	UP	^12^
miRNA-93	Up	^12^
Let-7	Down	^13^
miRNA-199a-3p	Down	^13^
miRNA-124	Down	^14^
miRNA-124-3p	Down	^14^
miRNA-29 a&c	Down	^14^
miRNA-26b-5p	Down	^15^
miRNA-200 a&b	Down	^15^
miRNA-607	?	none

The miRNAs reported in this Table showed the highest expression patterns for differentially expressed genes in all 6 pairwise comparisons reported previously. The majority of these miRNAs were found to be down regulated in the presence of HCC.

**Figure 6:**
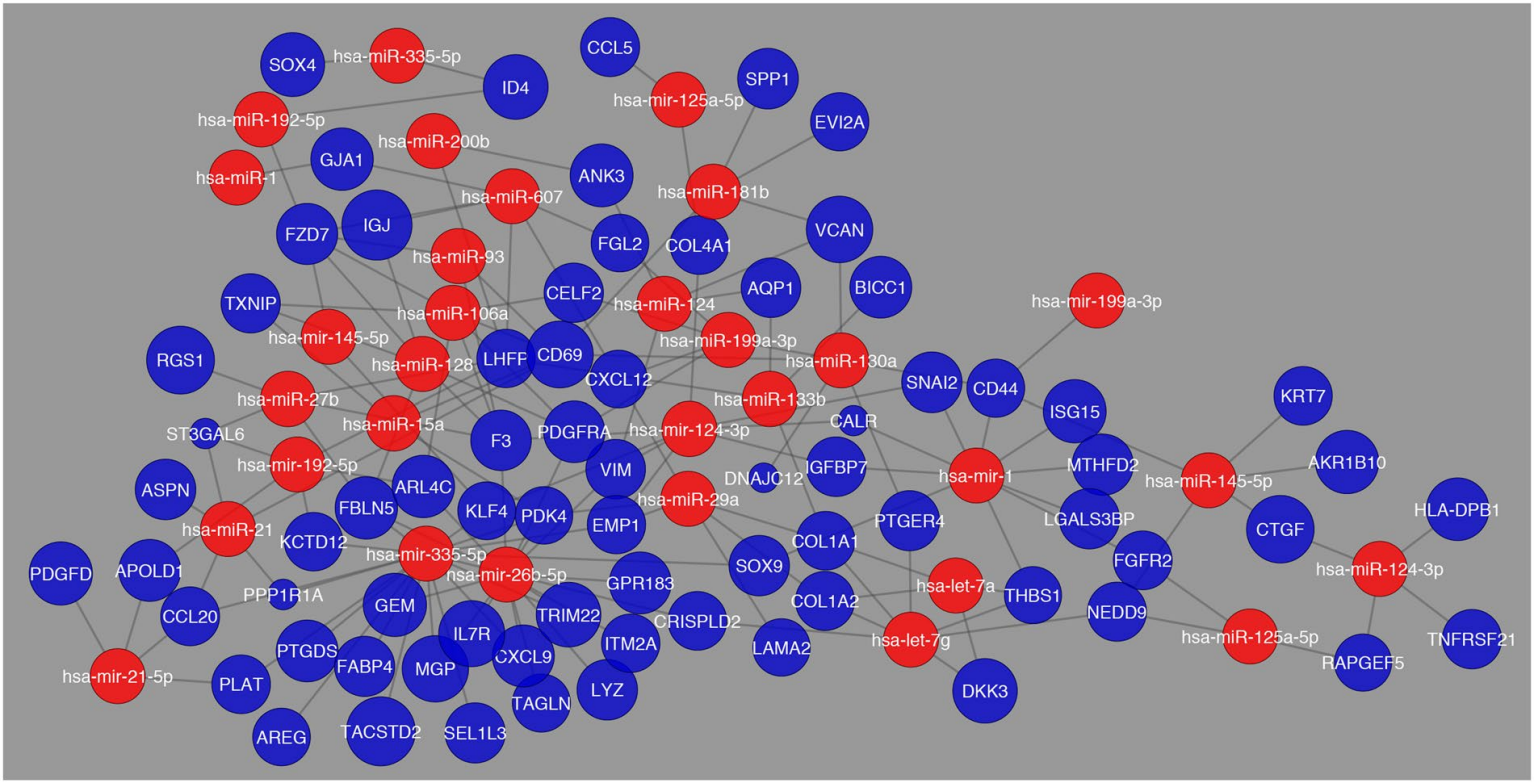
Network visualization of most common miRNAs regulating differentially expressed genes between Normal and Cirrhosis. The blue circles represent differentially expressed genes and their respective size is representative of their fold change value. The red circles represent the miRNAs that are regulating their respective differentially expressed genes.

The interaction between differentially expressed genes and the miRNAs with the highest expression pattern were constructed in a network for the comparison between Normal and Cirrhosis as shown in Fig. 6. Here it becomes apparent that there is a number of interactions that are occurring with no distinct interaction apparent.

**Figure 7:**
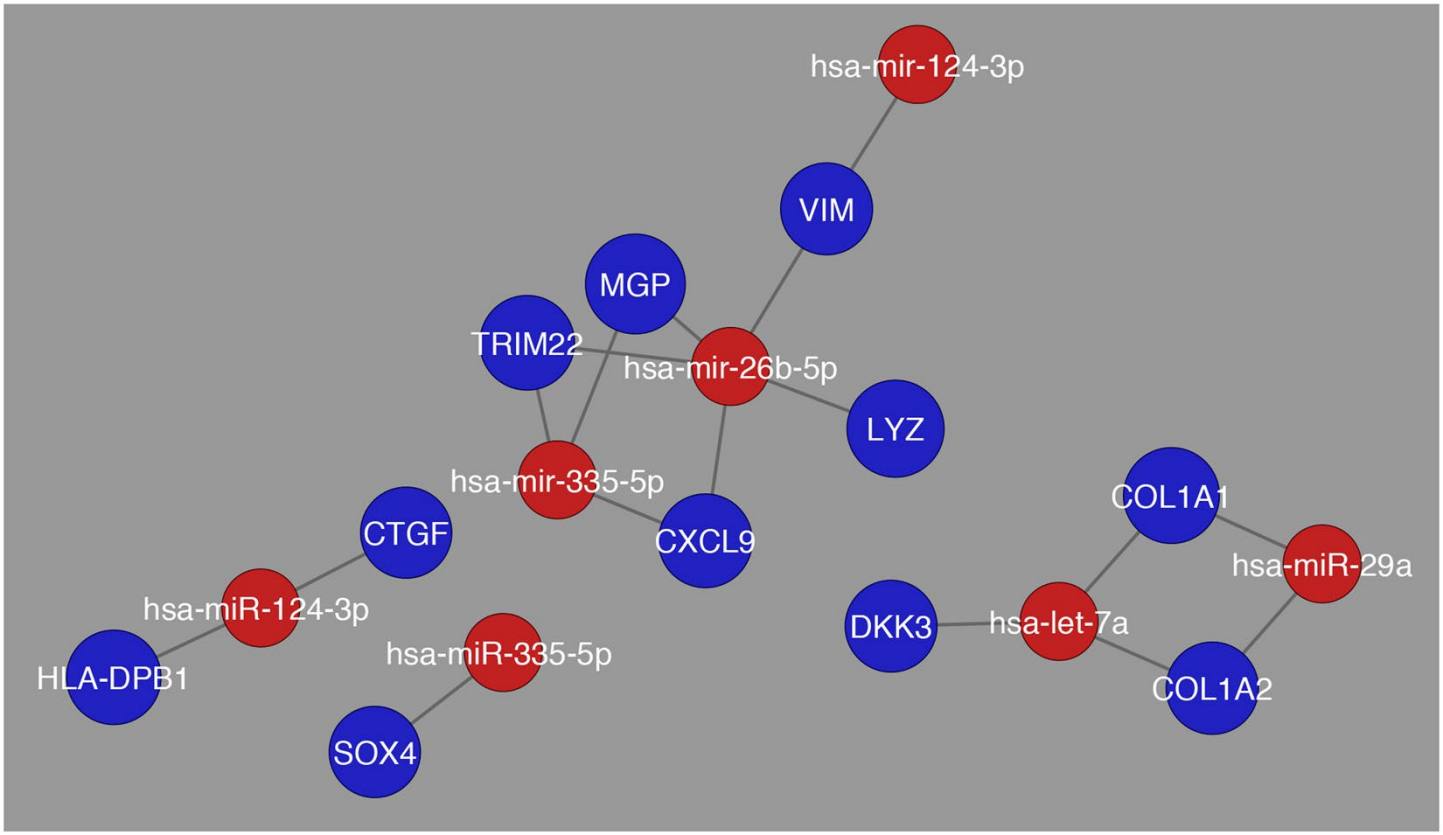
Network visualization of most common miRNAs regulating differentially expressed genes in the comparison between Normal and HCC. The blue circles represent differentially expressed genes and their respective size is representative of their fold change value. The red circles represent the miRNAs that are regulating their respective differentially expressed genes.

In this network (Fig. 6), the genes that were determined to have the greatest fold change were *MGP*, *TACSTD2*, *TRIM22*, and *CTGF*. This network differs from what was observed in the comparison between Normal and HCC, as shown in Fig. 7.

**Figure 8:**
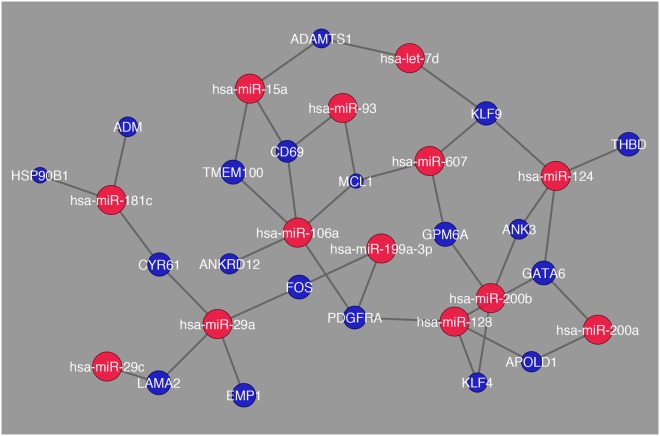
Network visualization of most common miRNAs regulating differentially expressed genes in the comparison between cirrhosis and HCC. The blue circles represent differentially expressed genes and their respective size is representative of their fold change value. The red circles represent the miRNAs that are regulating their respective differentially expressed genes.

In the comparison between Normal and HCC there are more distinct interactions apparent, along with less number of miRNAs showing high expression patterns. The genes that were determined to have the greatest fold change were *MGP*, *TRIM22*, *LYZ*, and *COL1A1*. Yet, the comparison between Cirrhosis and HCC revealed an even more distinct network than what was observed previously as shown in Fig. 8.

**Figure 9:**
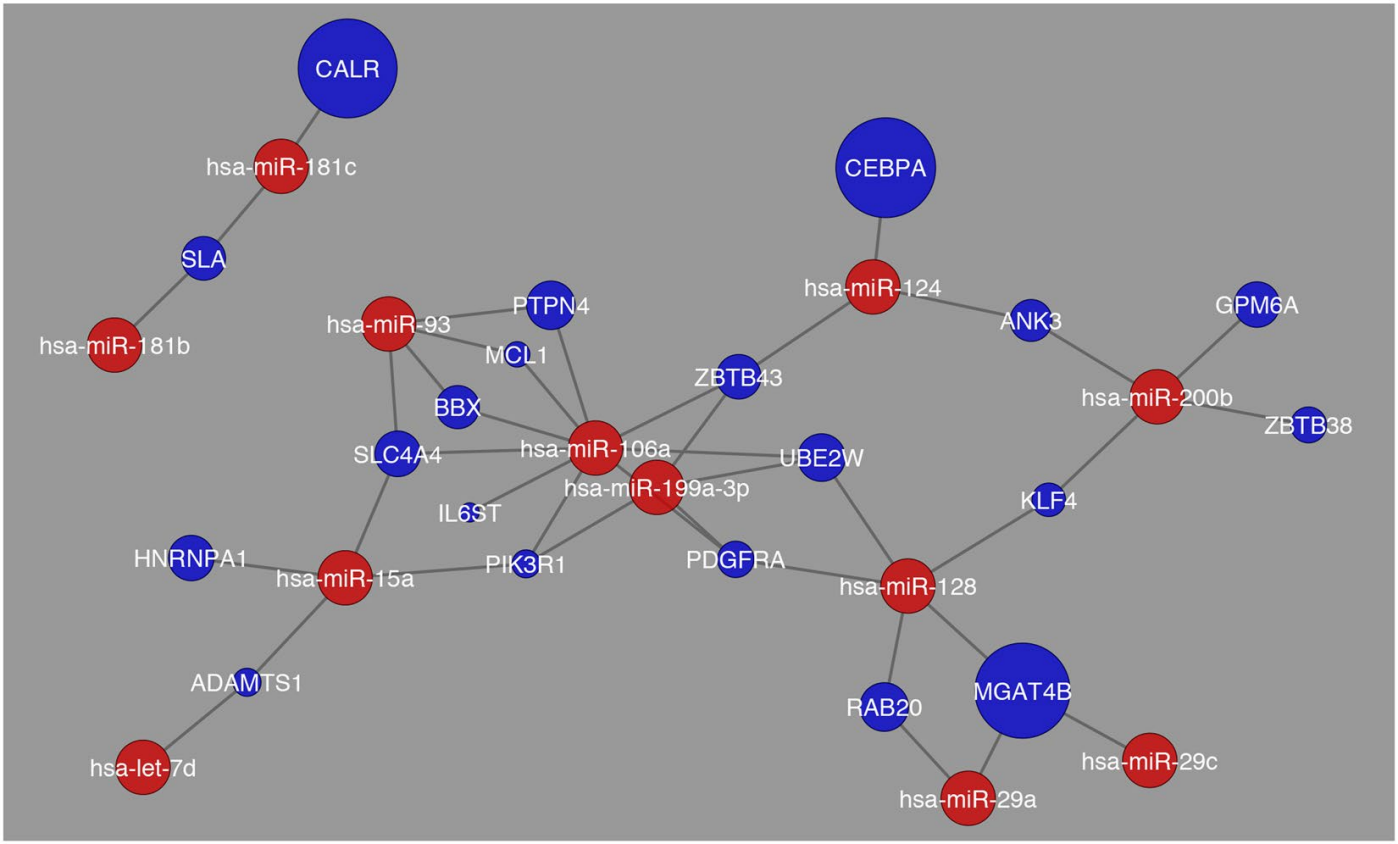
Network visualization of most common miRNAs regulating differentially expressed genes in the comparison between Cirrhosis HCC and HCC. The blue circles represent differentially expressed genes and their respective size is representative of their fold change value. The red circles represent the miRNAs that are regulating their respective differentially expressed genes.

**Figure 10:**
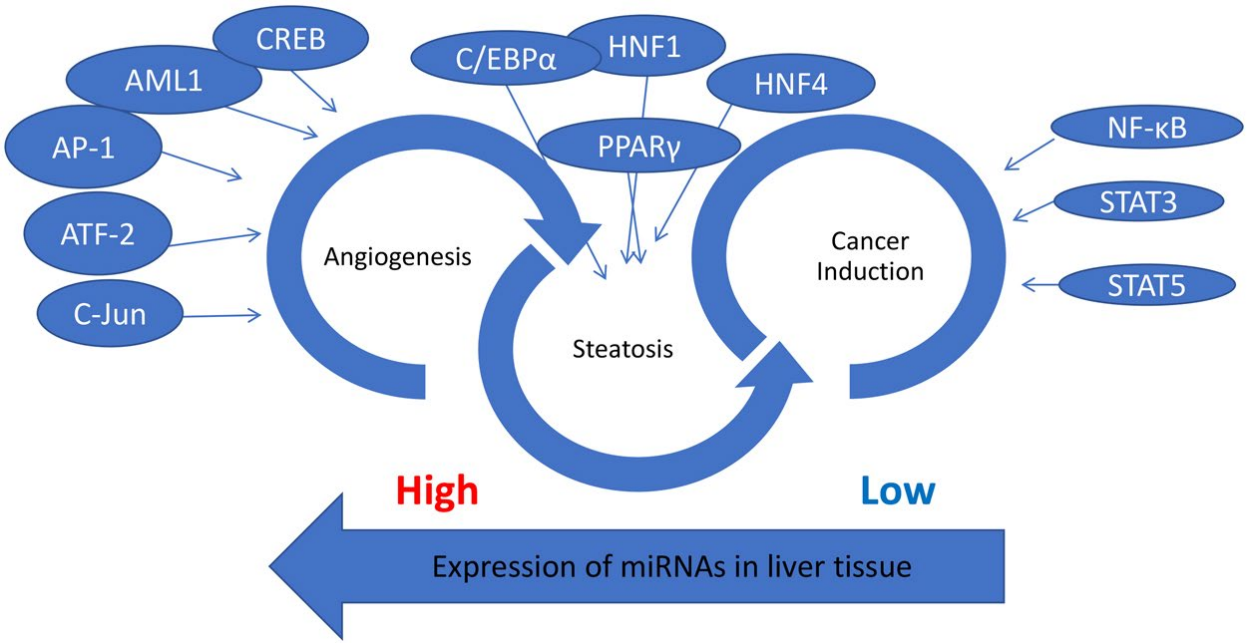
Overview of the transcription factors that regulate HCV induced HCC in each stage as the liver digresses to eventually becoming carcinomic; and the digression of miRNA expression in liver tissue as they digress to carcinoma.

For the network Cirrhosis_HCC there is an interaction between genes and miRNAs throughout the entire network along with a more distinct number of miRNAs and differentially expressed genes being regulated. Also, the number of genes that showed greatest fold change was *TMEM100* and *GPM6A*. This synchrony in interaction was not observed in the comparison between Cirrhosis HCC and HCC as shown in Fig. 9. While there are fewer interactions in this comparison there are still distinct interactions present as the number of genes that showed high fold change were greater than the previous comparison, and they were *CALR*, *CEBPA*, and *MGAT4B*. As a means of schematically outlining the findings, in Fig. 10, the transcription factors with the highest expression in all 6 pairwise combinations were further analyzed through a systematic analysis of the literature that is present and it was determined that these transcription factors are involved in regulating HCV induced HCC into three stages angiogenesis, steatosis, and cancer induction. Similarly, it was determined that the expression of miRNAs decreases as the liver digresses to carcinoma.

**Figure 11:**
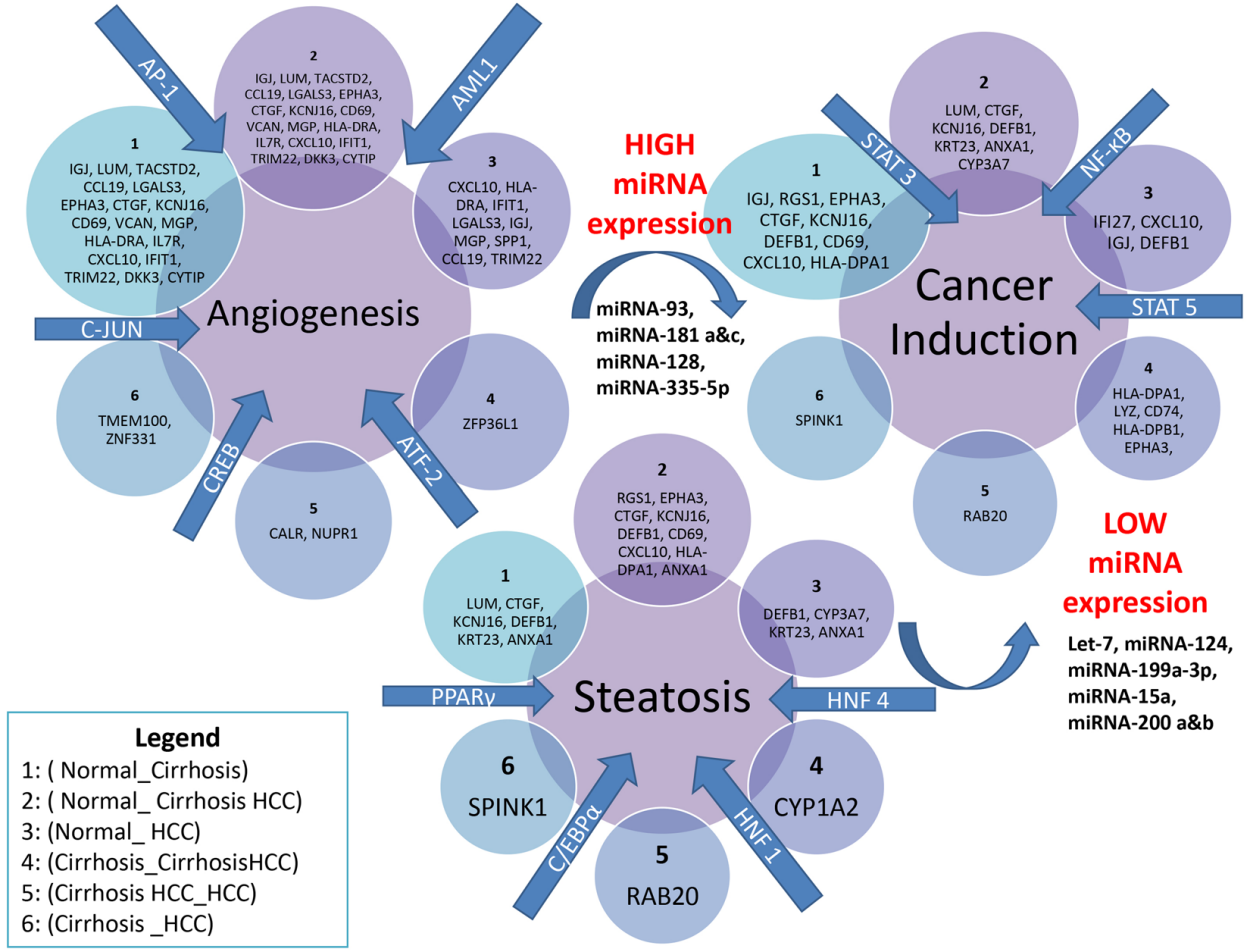
Transcription Regulation HCV-induced HCC. This is an illustration of the master regulators in HCV-induced HCC and their differentially expressed genes. The key genes and transcription factors associated with each condition of the liver is illustrated as they were found in each of the 6 pairwise comparisons, also with the general expression of miRNAs as the liver transitions from one condition to the next.

The differentially expressed genes regulated by their transcription factors in each stage in the digression of the liver are displayed in Fig. 11 so as to demonstrate the overall findings of this study and to illustrate the actions of all the key players in the transcription regulation of HCV- induced HCC.

## Discussion

### Differentially Expressed Genes

This study highlights the molecular switches which influence gene expression and may be involved in the progress of HCV-induced liver to HCC.

Analysis of unique probe sets for every condition revealed that the gene expression patterns varied significantly among the HCC and normal liver samples. These findings coincide with the findings of Mas and colleagues (2009), which showed that genes associated with cell proliferation and mitosis had increased expression in HCC samples, while those with lower expression are specifically expressed in differentiated hepatocytes^11^. In our findings, the DAVID analysis indicate that differentially expressed genes cluster, function in cell signaling. Also, the functionality of the genes does not change amongst the various pairwise comparisons between normal liver and cirrhosis as well as between cirrhosis and HCC. DAVID analysis determined that the primary functional clusters for differentially expressed genes in cirrhosis and HCC were involved in cell attachment, as well as cell division and apoptosis.

As the condition of the liver progresses to carcinoma the regulation of gene expression is determined not only by the condition of the liver but also by the presence of regulatory elements underlying the command of these genes such as transcription factors and miRNAs.

### Transcription Factors

As HCV infects more and more liver cells, the condition of the liver exacerbates, as the liver digresses from a cirrhotic liver, to steatosis, and may eventually become cancerous. Although, this sequence of events is not dogma and the liver may digress from any one of these conditions directly to carcinoma, if the attributes that satisfy the conditions for each event are present the liver will digress in this sequence and eventually become carcinomic. Each one of these conditions entails a different molecular key switch as different genes are turned on and off to substitute for the digressing functionality of the liver. Pairwise comparisons showed that cell signaling as well as other important cellular functions were of the most common functionalities effected as the condition of the liver digresses from normal to cancerous in the presence of HCV infection.

Of the keywords which arise in the functional annotation clustering tool (DAVID) is the keyword “fat”; which may indicate that the differentially expressed genes may also be linked to lipid metabolism. HCV infection relies heavily on lipid metabolism in the liver for viral entry and replication^12^. Assembly of HCV virion is brought about by the synchronized actions of HCV structural proteins along with the viral replication complex, and takes place in close proximity to membrane bound lipid droplets (LD)^13^. LDs are intracellular organelles storing cholesterol and triglycerides, which play an essential role in viral packaging. Inhibition of the synthesis of their lipid components blocks viral assembly. In the blood HCV virions circulate as lipo-viro-particles (LVPs) which are found to be associated with very low or low-density lipoproteins (VLDLs and LDLs). These LVPs may also associate with high levels of triglycerides, Apolipoprotein (Apo) E, ApoC, ApoB and low levels of cholesterol or phospholipids^14^. This HCV virion association with lipids may facilitate its infection of hepatocytes which are the primary target for infection while at the same time aiding in its escape from neutralizing antibodies^15^. This in turn would explain the association between differentially expressed genes and the keyword FAT in the functional annotation clustering of genes.

Annotation of the differentially expressed genes with their respective transcription factors coincides with the functional annotation of the differentially expressed genes in that it was found that main transcription factors involved in regulation of the differentially expressed genes were marked by 3 hepatic conditions; angiogenesis, steatosis, and carcinoma. In Fig. 4, it becomes apparent that many genes involved in normal liver function are being regulated by the transcription factors involved in each hepatic condition. Each of these hepatic conditions calls upon a different key switch of genes and transcription factors which foretells the possible points at which molecular intervention may be key to stop the digression of liver to carcinoma and possibly save the liver or freeze it in its current state.

Angiogenesis and the disruption of liver vascular architecture have been linked to progression to cirrhosis and Hepatocellular Carcinoma (HCC) in chronic liver diseases, which contributes both to increased hepatic vascular resistance and portal hypertension and to decreased hepatocyte perfusion^16^. Angiogenesis was also found to be important in adult tissue repair^17^. In the present study, 5 main transcription factors, *AP-1*, *C-JUN*, *AML1*, *CREB*, and *ATF-2*, were determined to have a common expression pattern amongst all 6 pairwise comparisons. These factors may be the key switches in the effect of transcriptional regulation of differentially expressed genes. These 5 transcription factors were reported to regulate genes involved in hepatic angiogenesis^18^.

Angiogenesis is implicated in cancer development by favoring progression, growth, and metastasis of cancer and is regulated by growth β factor-beta (*TGF-β*), basic fibroblast growth factor, or platelet-derived growth factor. In tumor cells induction of hepatic angiogenesis during chronic HCV infection may contribute to the early development of cancer cells^18^.

One of the 5 transcription factors acute myelogenous leukemia 1 *(AML1*)/runt-related transcription factor 1 (*Runx1*) belongs to a family of transcriptional regulators called *Runx*. AML1 is known to directly regulate cyclin D gene expression. Cyclin D proteins have been characterized as oncogenes. Cyclin D proteins are involved in the tumorigenesis of various human malignancies including HCC^19^.

Gene-targeting studies in mice have demonstrated that *AML1* is essential for early development of definitive hematopoiesis^20^. A study by Takakura and colleagues (2000) showed that *AMl1* deficient embryos lack definitive hematopoiesis and thus show defective angiogenesis in the head and pericardium^17^. In another study, HCV core protein was shown to play a role in the regulation of hepatic angiogenesis during the course of HCV infection and was able to trigger the production of both *TGF-β2* and *VEGF* proteins by multiple pathways including PKC, RB/E2F1, ASK1-JNK/p38, and ERK^18^. These pathways include transcription factors that were found to be the main transcription regulators of differentially expressed genes^18^. Of these pathways, the PKC pathway was found to turn on *CREB* and *ATF-2*, while ASK1-JNK/p38 was found to control *AP-1*. *AP-1* is an important target for the JNK signaling pathway and is composed of *Jun*, *Fos*, and related bZIP subunit^21^.

As the liver vascular architecture changes and the liver proceeds in the direction of fibrosis another condition starts to develop as well; hepatic steatosis. This condition may be in part as a reaction to the changes that are already taking place in the liver as the liver may call upon lipids for the increasing viral particles and replication that is present due to HCV infection. Rutkowski *et al*. (2008) suggest that one of the possible reasons behind steatosis is the suppression of a subset of metabolic transcription factors that regulate lipid homeostasis as a direct response to ER homeostasis^22^. The exact mechanism of this digression from hepatic angiogenesis to hepatic steatosis is unknown, however it is apparent that each condition hampers the functionality of the liver leading to carcinoma.

Hepatic steatosis is common amongst HCV patients. HCV core protein was shown to induce the transcriptional activity of *PPARγ* (peroxisome proliferators-activated receptor γ)^23^. *PPARγ* as well as *HNF1*, *HNF4*, and *C/EBPα* were found to be amongst the transcription factors with the highest expression patterns regulating differentially expressed genes in each of the 6 pairwise comparisons involved in this study.

*PPARγ* is a master regulator for adipocyte differentiation and is important in the regulation of a number of genes involved in fatty acid and glucose metabolism. Thus down regulation of liver *PPARγ* which contributes to regulation of lipid synthesis, transport, and storage within hepatocytes, causes the development of hepatic steatosis^24^. CCAAT/enhancer-binding protein alpha (*C/ebpα*) another transcriptional regulator found to have high expression pattern in differentially expressed genes has been shown to be involved in regulating gluconeogenesis and lipogenesis while being downregulated in response to hepatic steatosis.

Hepatocyte nuclear factors 1 and 4 (*HNF1 and HNF4)* are a group of transcription factors that are involved with glucose, cholesterol, and fatty acid transport and metabolism^25^. HCV induces *HNF1* and *HNF4* due to increased oxidative stress and direct protein-protein interactions between HCV non-structural component (NS) 5A and *HNF1*. Again, some HCV proteins, particularly the structural capsid protein, core, and the non-structural protein, NS5A, can induce hepatic steatosis by interfering with intracellular lipid metabolism^26^. Alteration of expression of these genes might be related to abnormal expression of metabolic enzymes again leading to hepatic steatosis^27^.

Univariate and multivariate analyses identified hepatic steatosis, together with aging, cirrhosis, and lack of IFN treatment, as significant independent risk factors for HCC^28^. Several studies have revealed that hepatic steatosis, including steatosis induced by HCV core protein, predisposes to lipid peroxidation and excess free-radical activity with the potential risk of genomic mutations. Hepatic steatosis is an independent risk factor for HCC in patients with chronic HCV infection, although the factors responsible for steatosis could not be identified clearly^29^. However, a recent study has shown a novel link between obesity-induced lipid accumulation and selective CD4+T lymphocyte loss, suggest a critical role for CD4+ T lymphocytes in the disease progression from NAFLD to HCC^30^.

The production of a great number of cytokines, chemokines and growth factors, favoring increased cellular proliferation accompanies the continuous cell death and inflammatory cell infiltration during cancer development. This continuing hepatocyte death triggers liver repair and regeneration and eventually leads to severe liver fibrosis or cirrhosis^31^. Multiple signaling pathways are involved in this injury-inflammation-regeneration response and in human HCC development. In the present study 3 transcription factors were found to have high expression patterns amongst all 6 pairwise comparisons as a result of prolonged HCV infection; these transcription factors include *NF-κB*, *STAT3*, and *STAT 5*.

Nuclear factor kappa-light-chain-enhancer of activated B cells (*NF-κB)* is a protein complex that regulates cell survival, immunity and inflammation, and is one of the more important pathways that is activated during liver injury and inflammation. One of the main functions of *NF-κB* in hepatocytes appears to be the production of cytokines that maintain the inflammatory microenvironment in which tumors develop^6^.

Signal transducer and activator of transcription 3 (*STAT3*) is another transcriptional factor involved in immune responses, inflammation and tumorigenesis, and was found to be critical for compensatory liver regeneration and chemically-induced HCC development^6^. *STAT3* belongs to the signal transducer and activator of transcription (STAT) family. Like its relatives, *STAT3* is inactive in non-stimulated cells, but is rapidly activated by various cytokines and growth factors, such as IL-6 and EGF family members, as well as hepatocyte growth factor (*HGF*) *STAT3* in cancer cells is activated by cytokines and growth factors that are produced within the tumor microenvironment. The expression of IL-6, one of the major *STAT3*-activating cytokines, and is elevated in human liver diseases and HCC^32^.

Signal transducer and activator of transcription 5 (*STAT5*) proteins are involved in cytosolic signaling and in mediating the expression of specific genes. While *STAT5* activation plays an important role in promoting tumorigenesis via the upregulation of anti-apoptotic, cell proliferative, and invasion of metastasis-related genes^32^. In HCC Stat5 activation is associated with advanced tumor stages. Stat5 enhances HCC aggressiveness through induction of epithelial–mesenchymal transition. However, loss of Stat5 in mice caused steatosis, liver fibrosis and promoted chemically induced liver cancer. This was partly explained by a compensatory pYStat1/ pYStat3 and TGF-β axis, where a new role for the Stat5 N-terminus was described in binding directly TGF-β^33^.

### miRNAs

The molecular mechanisms underlying transcriptional regulation of miRNA genes in the liver remain largely unknown. Whether transcription factors are also involved in the transcriptional regulation of miRNAs in the liver is unclear since the functional expression of transcription factors can also be regulated by miRNAs. Yet, it is clear that miRNAs can potentially regulate every aspect of cellular activity and a diverse spectrum of liver functions^34^.

In the present study, it was observed that each tissue type or condition had its own respective signature miRNA pattern, however there was a commonality amongst all 6 pairwise comparisons that were analyzed as shown in the Table 1. As was observed for the expression of genes regulated by the master transcription factors involved in each hepatic condition, the number of genes that are regulated by miRNAs decreases as the number of genes involved in normal liver function decreased. However, unlike transcription factors, miRNAs did not show a functional relationship between the genes that they regulate; again, this reflects the specificity by which miRNAs regulate the expression of genes. In Table 2 the miRNAs that had the highest expression were annotated with their predicted differentially expressed target genes in all 6 pairwise comparisons. After analysis using functional annotation clustering it becomes apparent that the differentially expressed genes that miRNAs regulate are involved in maintaining cellular homeostasis and the up keeping of fat metabolism. Therefore, as cellular homeostasis becomes imbalanced and fat metabolism is disrupted the expression of miRNAs decreases as the liver digresses to carcinoma.

**Table 2:** The miRNAs with the highest expression in all 6 pairwise combinations were annotated with their differentially expressed target genes.

miRNAs	Predicted Target Genes
Let-7	*THBS1*, *COL1A2*, *COL1A1*, *DKK3*, *ADAMTS1*, *KLF9*
miR-124-3p	*HLA-DPB1*, *COL4A1*, *TNFRSF21*, *CTGF*, *RAPGEF5*, *CALR*, *EMP1*, *IGFBP7*, *SNAI2*, *VIM*, *F3*, *KLF4*, *EMP1*, *IFI44L*, *MFAP4*, *RBPMS*
miR-124	*ANK3*, *AQP1*, *VCAN*, *VIM*, *THBD*, *KLF9*, *GATA6*, *ZBTB43*, *CEBPA*
miR-29a	*LAMA2*, *EMP1*, *PDK4*, *COL1A2*, *COL1A1*, *CXCL12*, *ENG*, *CYR61*, *RAB20*, *MGAT4B*
miR-29c	*LAMA2*, *COL1A2*, *COL1A1*, *COL6A2*, *LAMA2*, *MGAT4B*
miR-26b-5p	*GEM*, *ITM2A*, *MGP*, *FBLN5*, *CXCL9*, *GPR183*, *ARL4C*, *LYZ*, *PDK4*, *GPR183*, *F3*, *CCL2*, *PDGFRA*, *TRIM22*, *VIM*, *TAGLN*, *FABP4*, *IFI44L*, *VIM*, *ARL4C*, *IL7R*
miR-335-5p	*TRIM22*, *PDK4*, *SOX9*, *PTGDS*, *CRISPLD2*, *CXCL9*, *PPP1R1A*,*TACSTD2*, *MGP*, *CCL20*, *FABP4*, *GEM*, *FBLN5*, *AREG*, *PLAT*, *EMP1*, *SEL1L3*, *KCTD12*, *SOX4*, *ID4*, *SH3YL1*,
miR-27a&b	*ST3GAL6*, *FBLN5*, *RGS1*, *LHFP*, *F3*
miR-106a	*ARL4C*, *FZD7*, *PDGFRA*, *TXNIP*, *F3*, *CD69*, *CELF2*, *ANKRD12*, *TMEM100*, *CD69*, *MCL1*, *IL6ST*,*PIK3R1*,*PDGFRA*, *UBE2W*, *ZBTB43*, *PTPN4*, *MCL*, *BBX*, *SLC4A4*
miR-128	*PDGFRA*, *KLF4*, *APOLD1*, *UBE2W*, *PDGFRA*, *RAB20*, *MGAT4B*
miR-15a	*TXNIP*, *PDK4*, *CD69*, *EIF4B*,*TMEM100*, *ADAMTS1*, *HNRNPA1*, *ADAMTS1*, *SLC4A4*, *PIK3R1*
miR-181a	*CD69*, *VCAN*, *SPP1*, *EVI2A*
miR-181c	*CYR61*, *HSP90B1*, *ADM*, *CALR*, *SLA*
miR-199a-3p	*PDGFRA*, *CELF2*, *CXCL12*, *FGL2*, *CD44*, *KRT7*, *ZBTB43*, *PIK3R1*, *UBE2W*, *FOS*
miR-200a	*APOLD1*, *GATA6*,
miR-200b	*LHFP*, *ANK3*, *GEM*, *ADAMTSL2*, *KLF4*, *GPM6A*, *ZBTB38*,
miR-93	*F3*, *CD69*, *FZD7*, *TGFB1I1*, *VIM*, *MCL1*, *BBX*, *SLC4A4*, *PTPN4*
miR-607	*FZD7*, *IGJ*, *FGL2*, *CXCL12*, *LHFP*, *GJA1*

Of the miRNAs that were found to be downregulated miR-27, miR-199, miR-200, and let-7 family members were amongst them and were found to target genes involved in cell cycle and cell death regulation^35^. These miRNAs were also found in this study to have high expression in normal liver tissue and eventually no expression in carcinoma tissue with HCV infection. Downregulation of the miRNAs miR-26, miR-29, and miR-124 have been implicated in cell proliferation, apoptosis, angiogenesis, and poor prognosis^36^. In a previous study, a correlation between miR-106a and the degree of differentiation was shown to suggest an involvement of specific miRNAs in the progression of the disease^34^. Lee and colleagues provide data to support the down-regulation of miR15a and increased cellular proliferation^36^.

MiRNAs that were found to be upregulated were miRNA-335-5p, miR-93, miR-181a & c, and miR-128. MiRNA-335-5p was found to be associated with non-alcoholic fatty liver disease and also showed a connection with metabolic disorders^35^. MiR-93 is overexpressed in HCC and may have a critical role in cell proliferation by regulating the G1-to-S cell cycle transition^35^. MiR-181 is upregulated and promotes migration and invasion of HCC cells^37^. The up-regulated miR-128, has nearly perfect complementarity in its seed sequences with HCV RNA genomes and is capable of inhibiting HCV replication and infection^34^.

As for miR-607 while it was of the miRNAs with high expression patterns for differentially expressed genes there was no information in the literature to date as to how it influences the digression in the condition of the liver or even how it regulates genes in the development of HCC. However, it can be noted that mIR-607 does act as a tumor suppressor in other cancers, such as male breast cancer^38^.

In this study it was apparent that a plethora of miRNAs may underlie the control of cellular homeostasis in the liver. It was also apparent that each liver condition had its signature expression pattern of miRNAs. Yet, the miRNAs that were found to have differential expression in all 6 pairwise comparisons were shown to be master regulators in transcriptional regulation and in turn have an impact on the conditions of the liver and the progression to HCC. Understanding the molecular mechanisms that underlie the influence HCV on the progression to HCC would aid the development of effective treatments. The master transcriptional regulators that dictate each stage in the progression of HCV infected liver to HCC were determined. Understanding the molecular mechanisms that underlie the influence HCV on the progression of liver to HCC would aid the development of effective treatments.

## Methods

### Dataset

Samples used in this study were obtained from the Gene Expression Omnibus (GEO) with the identification number GSE14323. From this database, 124 samples were downloaded in the form of a compressed file (500MB), extracted, and prepared for further processing in the form of .cel files^11^. This dataset was used in particular because it provided a sample pool that would allow for a distinct look at the transcriptional control of genes. It provides a clear stage by stage tissue sample for analysis and comparison^11^.

### Sample Clusters Compared

In order to study transcription regulation, the differential expression in each of the four sample clusters (normal, cirrhosis, cirrhosis with HCC, and HCC) were compared in pairs using R studio, and the following six comparisons: Normal vs. Cirrhosis, Normal vs. Cirrhosis HCC, Normal vs. HCC, Cirrhosis vs. Cirrhosis HCC, Cirrhosis vs. HCC, and Cirrhosis HCC vs. HCC; were made.

### Data Analysis

Samples included 124 liver tissue samples obtained from 88 distinct HCV patients, 41 HCV-cirrhotic from patients without HCC, 17 cirrhotic tissues from patients with HCC, and 47 HCV-HCC tissues. Also, 13 patients with HCC-cirrhosis provided both tumor and cirrhotic tissue, 3 patients provided both cirrhotic or tumor tissue for array processing. Nineteen normal liver tissues were included from individuals with normal liver function and histopathology, and seronegative for HCV Ab^11^.

The samples that were obtained were further processed using R version 3.2.0. Samples in the form of .cel files were found to be in two different Affymetrix platforms because of the difference in probes used for the samples. Therefore, in R studio an affy bioconductor package was downloaded to read the samples in the form of .cel files and samples were separated into DATA1 and DATA2 based on the Affymetrix platform corresponding to the samples. These samples were converted from. cel files which are files that correspond to light intensities into .cdf files that converted light intensities into probe concentrations using the affy Bioconductor package in R studio. After which these values were then converted into expression values using the Robust Multiarray Average (RMA). These expression values were then saved for further processing.

In order to analyze the dataset for differentially expressed genes, the samples were further analyzed using R version 3.2.0. Since this study contained 124 samples, these samples were grouped into 4 matrices: normal, cirrhosis, cirrhosis HCC, and carcinoma.

Once the differentially expressed genes were determined for each case, they were manually paired with their corresponding transcription factors and miRNAs. Using various databases such as TRANSFAC, miRBASE, and GENECARDS, transcription factors and miRNAs were searched for in order to correspond to genes that were determined to be differentially expressed. These results were then written into a table in the form of a text file.

Tables that were created with differentially expressed genes and their corresponding transcription factors and miRNAs were evaluated using Cytoscape version 3.2.1. Tables were imported in the form of text files and a network was created in order to visualize the structure of how the genes are connected to their transcription factors and miRNAs. In each network genes are colored in blue, transcription factors are in green, and miRNAs are in red. The size of genes corresponded to their fold change, and each network was analyzed and clusters were determined, if any.

Differentially expressed genes were also functionally annotated using DAVID database. This database clustered genes in each case based on their function. Genes were delivered to the database in the form of a list, case by case, and the database clustered the list of genes based on the similarity of the genes functionally. A schematic diagram (Fig. 1) of the study was produced in order to visually understand the procedures by which this study was conducted.

## Data Availability

The datasets generated during and/or analyzed during the current study are available from the corresponding author upon request.
